# Enhanced CT-based radiomics predicts pathological complete response after neoadjuvant chemotherapy for advanced adenocarcinoma of the esophagogastric junction: a two-center study

**DOI:** 10.1186/s13244-022-01273-w

**Published:** 2022-08-17

**Authors:** Wenpeng Huang, Liming Li, Siyun Liu, Yunjin Chen, Chenchen Liu, Yijing Han, Fang Wang, Pengchao Zhan, Huiping Zhao, Jing Li, Jianbo Gao

**Affiliations:** 1 Department of Radiology, The First Affiliated Hospital of Zhengzhou University, Zhengzhou, China; 2 Pharmaceutical Diagnostics, GE Healthcare (China), Beijing, China; 3 Department of Radiology, The Affiliated Cancer Hospital of Zhengzhou University (Henan Cancer Hospital), Zhengzhou, China

**Keywords:** Esophagogastric junction, Adenocarcinoma, X-ray computed tomography, Neoadjuvant therapy, Response evaluation

## Abstract

**Purpose:**

This study aimed to develop and validate CT-based models to predict pathological complete response (pCR) after neoadjuvant chemotherapy (NAC) for advanced adenocarcinoma of the esophagogastric junction (AEG).

**Methods:**

Pre-NAC clinical and imaging data of AEG patients who underwent surgical resection after preoperative-NAC at two centers were retrospectively collected from November 2014 to September 2020. The dataset included training (*n* = 60) and external validation groups (*n* = 32). Three models, including CT-based radiomics, clinical and radiomics–clinical combined models, were established to differentiate pCR (tumor regression grade (TRG) = grade 0) and nonpCR (TRG = grade 1–3) patients. For the radiomics model, tumor-region-based radiomics features in the arterial and venous phases were extracted and selected. The naïve Bayes classifier was used to establish arterial- and venous-phase radiomics models. The selected candidate clinical factors were used to establish a clinical model, which was further incorporated into the radiomics–clinical combined model. ROC analysis, calibration and decision curves were used to assess the model performance.

**Results:**

For the radiomics model, the AUC values obtained using the venous data were higher than those obtained using the arterial data (training: 0.751 vs. 0.736; validation: 0.768 vs. 0.750). Borrmann typing, tumor thickness and degree of differentiation were utilized to establish the clinical model (AUC-training: 0.753; AUC-validation: 0.848). The combination of arterial- and venous-phase radiomics and clinical factors further improved the discriminatory performance of the model (AUC-training: 0.838; AUC-validation: 0.902). The decision curve reflects the higher net benefit of the combined model.

**Conclusion:**

The combination of CT imaging and clinical factors pre-NAC for advanced AEG could help stratify potential responsiveness to NAC.

**Supplementary Information:**

The online version contains supplementary material available at 10.1186/s13244-022-01273-w.

## Key points

- Radiomics method can predict that AEG patients can achieve pCR after NAC.
- The combination of radiomics and clinical factors can improve the predicting performance.
- Radiomics–clinical model can stratify patients according to potential responsiveness to NAC.
- Radiomics–clinical model can help clinicians to develop individualized and precise treatment plans.

## Introduction

The incidence of adenocarcinoma of the esophagogastric junction (AEG) has been increasing in recent years [1]. AEG is defined as adenocarcinoma with a tumor center located within 5 cm above or below the anatomical esophagogastric junction (EGJ) that crosses or touches the EGJ. Radical surgical resection and complete lymph node dissection are the basis of treatment, but most patients with AEG are already in the advanced stage when diagnosed, and surgical resection alone has a high rate of local recurrence and poor survival [2]. Neoadjuvant chemotherapy (NAC) followed by radical surgery has become a recommended treatment modality for advanced gastric cancer patients. NAC can reduce tumor load, lower tumor stage, improve the rate of radical resection and ultimately may prolong the survival of AEG patients [3–5]. However, the prognostic improvement that is achieved using NAC is largely dependent on the pathological response of the lesion after chemotherapy. The histological tumor regression grade (TRG) is an objective indicator used to evaluate the efficacy of therapy for AEG patients when cancer cells in the lesion are absent or when only a small number remain [6, 7]. In patients with a mild pathological response or poor response, NAC not only does not benefit the patient, but there is also a risk for toxic effects during chemotherapy and tumor progression, and delayed surgery may result in a decreased rate of radical resection and increased surgical complication rates [3, 8–11]. The current evaluation of the efficacy of NAC is based on the response evaluation criteria in solid tumors (RECIST) [12]. However, the indeterminate wall morphology of the GI tract and the fibrotic changes caused by NAC limit the application of RECIST; additionally, RECIST cannot be used to predict treatment response before treatment, and thus, there is a delay before it can be used [13–18]. Therefore, it is crucial to evaluate the efficacy of NAC in a timely and accurate manner before treatment to enable early identification of patients who will respond to NAC, and so that those who will respond poorly can immediately receive surgical treatment. Such an approach could help develop individualized treatment plans for AEG patients, improve patient prognosis and save valuable medical resources.

Along with traditional CT-based response evaluation, the radiomics method has great potential for revealing deep imaging information. The method adopts a combined medical-industrial approach to transform traditional images into deep-level digital quantitative features; the technique can be used to uncover the potential biological characteristics and heterogeneity within the tumor using the imaging data and to provide information beyond the morphological and functional characteristics of the tumor [19–22]. Several radiomics-based studies regarding the neoadjuvant treatment of esophageal and gastric cancers have been reported [3, 4, 23–25]. However, the use of this technique for the pre-NAC prediction of NAC response for AEG alone is rarely reported in most clinical studies of esophageal or gastric cancers, and most studies are single-center studies that lack independent external validation [26].

The purpose of our study was to retrospectively analyze the clinical, pathological and enhanced CT imaging data of patients with advanced AEG before NAC. The models used for predicting pathological complete response (pCR) to NAC were developed and externally validated. We hope the study will provide evidence for understanding tumor response-related quantitative imaging characteristics and contribute to the development of individualized and precise treatment strategies for AEG patients.

## Materials and methods

The study protocol was approved by the Medical Ethics Committee of Zhengzhou University, and the need for informed consent was waived.

### Patient selection

Clinical, pathological and CT imaging data of AEG patients who underwent surgical resection after preoperative NAC at the First Affiliated Hospital of Zhengzhou University and Henan Provincial Cancer Hospital were retrospectively collected from November 2014 to September 2020. The patient enrollment criteria included the following: (1) AEG confirmation by gastroscopic biopsy pathology prior to treatment; (2) pre-NAC clinical stage of cT_2_–_4_N_0_–_3_M_0_ stage; (3) NAC treatment in 2–6 cycles; (4) lack of other antitumor therapy administered before NAC; (5) enhanced CT scan obtained within 1 week prior to NAC treatment with complete imaging data; (6) lesion covering at least 3 slices on CT cross section and a maximum plane diameter of at least 2 cm; and (7) radical resection performed after NAC treatment with complete postoperative pathology data. The exclusion criteria included: (1) combined history of other malignancies; (2) poor CT image quality or lack of raw DICOM data; (3) adverse event during NAC or less than 2 cycles of NAC; (4) combined heart, lung and other important organ dysfunction in which a CT examination could not be performed; and (5) incomplete CT imaging data or clinical and pathological data.

Patients with AEG from the First Affiliated Hospital of Zhengzhou University were included in this study as the training group (*n* = 60), and 32 patients with AEG from Cancer Hospital of Zhengzhou University were included as the external validation group (*n* = 32).

### Clinical data

The chemotherapy regimens included the following: (1) XELOX regimen: oxaliplatin given intravenously at 130 mg/m^2^ for 2 h on Day 1, repeated every 3 weeks; patients received capecitabine at a dose of 1000 mg/m^2^ (bid, 1–14 d) orally twice daily; (2) SOX regimen: oxaliplatin 130 mg/m^2^ intravenously + oral capsules 80 mg/m^2^ in combination for 14 days twice daily. Patients and families signed informed consent forms. The treatment consisted of between 2 and 6 cycles unless disease progression, intolerable toxicity, or death occurred. All patients underwent radical surgical resection within 1 week of the end of NAC treatment.

The clinical data collected in our study included age, sex, carcinoembryonic antigen levels (CEA, normal range 0–5 ng/mL), carbohydrate antigen 199 level (CA199, normal range 0.01–37 U/ml), carbohydrate antigen 125 levels (CA125, normal range 0.01–35 U/ml), and serum albumin levels (normal range 35–55 g/l). TNM staging of tumors performed using CT images was evaluated according to the 8th edition of the American Joint Committee on Cancer (AJCC) staging [27]. The Borrmann typing of the AEG was documented [28]. The postoperative pathological TRG grading was recorded as a criterion for evaluating the efficacy of NAC. A TRG grade of 0 is defined as complete response, with no viable cancer cells remaining in the primary lesion and lymph nodes; TRG grade 1 is defined as moderate response, with single or small clusters of cancer cells remaining in the lesion; TRG grade 2 is defined as mild response, with significant disappearance of cancer cells under the microscope but some amount of cancer cells remaining, but with less interstitial fibrosis; and TRG grade 3 is defined as poor response, with no significant disappearance of cancer cells under the microscope or only a few cancer cells remaining. Patients were divided into the pCR group (tumor regression grade [TRG] = grade 0) and the nonpCR group (TRG = grade 1–3) based on the postoperative pathological histological TRG evaluation. In the training group, there were 19 patients in the pCR group and 41 patients in the nonpCR group. In the external validation group, there were four patients in the pCR group and 28 patients in the nonpCR group. The clinical characteristics of the enrolled patients are summarized in Table 1.

**Table 1:** Clinical characteristics of the enrolled patients in the training group and the external validation group

	Training group (*n* = 60)	Validation group (*n* = 32)	*p* value
*Gender*			
Male	49	24	0.993
Female	11	8	
Age (years)	60.60 ± 9.33	62.50 ± 5.86	0.340
Serum CA125 (Elevated)	6	1	0.300
Serum CA199 (Elevated)	8	5	0.241
Serum CEA (Elevated)	18	9	0.112
Serum albumin (Reduced)	29	1	0.315
*Borrmann typing*			
I–II	32	10	0.010*
III–IV	28	22	
Tumor thickness (cm)			
	18.19 ± 5.75	15.39 ± 5.14	0.036*
*Degree of differentiation*			
Low	25	20	0.087
Middle-high	35	12	
*T-staging before NAC*			
4	31	21	0.122
2–3	29	11	
*N-staging before NAC*			
0–1	38	17	0.578
2–3	22	15	
*TRG*			
0	19	4	0.218
1	12	10	
2	15	9	
3	14	9	

*NAC* neoadjuvant chemotherapy, *TRG* tumor regression grade, *CA* carbohydrate antigen, CA199 (normal range 0.01–37 U/mL), CA125 (normal range 0.01–35 U/mL), *CEA* carcinoembryonic antigen (normal range 0–5 ng/mL)

*Statistically significant level: *p* < 0.05

### CT image acquisition

All patients underwent contrast-enhanced CT scans, and informed consent forms were signed before inspection. The CT scans were acquired with a 64-row CT scanner (Discovery CT 750 HD, GE Healthcare, Waukesha, WI, United States) or a 256-row CT scanner (Revolution CT, GE Healthcare, Waukesha, WI, United States). Preparation for the examination occurred as follows: Patients fasted for more than 8 h before the examination and were given an intramuscular injection of scopolamine 10–20 mg 15–20 min before the examination to reduce gastrointestinal motility (Hangzhou Minsheng Pharmaceutical PG Roup Co., Ltd., Specifications: 10 mg/ml) and breath-holding exercises were performed. The patients also drank 800–1000 mL of warm water 10–15 min before the examination. The scanning parameters were as follows: tube voltage 120 kV; tube current using automatic milliampere second technology with a pitch of 1.375/1.1; field of view (FOV) of 500 mm; matrix 512 × 512 mm; and a scan thickness of 0.625–5 mm with scan spacing from 0.625 to 5 mm. The scan area at least encompassed the lower esophagus to the lower border of both kidneys. For the enhancement scan, 90–100 mL of nonionic contrast agent was injected through the elbow vein using a high-pressure syringe (iopromide, 370 mg/mL, GE Medical Systems, 1.5 mL/kg and 3 mL/s). Using the low-dose trigger technique, when the descending aorta reached 100 HU after the injection of contrast medium, arterial phase images were collected 10 s later, and venous phase images were collected at intervals of 30 s.

### Image processing and ROI segmentation

**Fig. 1:**
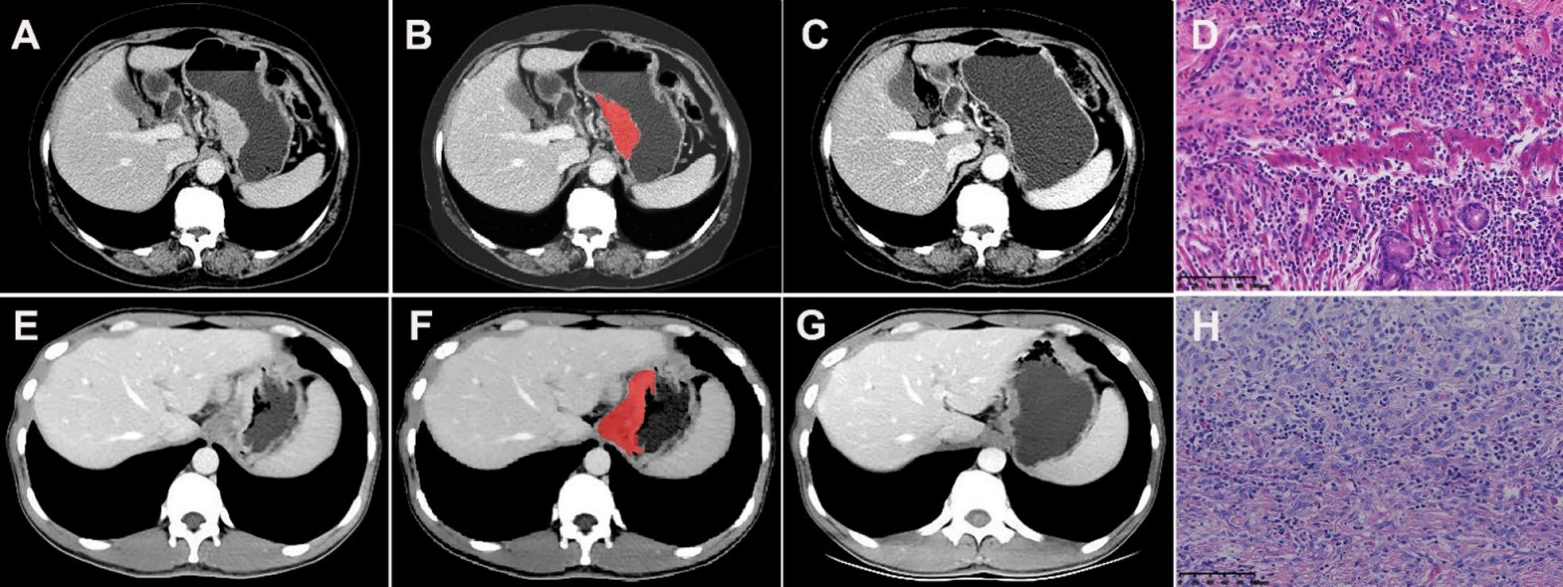
Hypofractionated adenocarcinoma of the esophagogastric junction (AEG) in a 63-year-old man. **A** CT venous phase axial image before neoadjuvant chemotherapy (NAC), Borrmann staging type I, thickest tumor diameter of 3.4 cm. **B** Schematic diagram of region of interest (ROI) segmentation on ITK-SNAP software. **C** CT venous phase axial image after NAC, lesion near disappearance, and insignificant gastric wall thickening. **D** Postoperative pathological images; fibrous tissue hyperplasia with chronic inflammatory cell infiltration was seen; no tumor cells remained; and tumor regression was obvious (HE × 200). Hypofractionated AEG in a 36 years old man. **E** CT venous phase axial image before neoadjuvant chemotherapy (NAC), Borrmann staging type III, thickest tumor diameter of 3.2 cm. **F** Schematic diagram of region of interest (ROI) segmentation on ITK-SNAP software. **G** CT venous phase axial image after NAC, significantly smaller lesions with reduced enhancement. **H** Postoperative pathological images showed more chronic inflammatory cell infiltration in the mucosal and lamina propria layers with focal fibrosis (HE × 200)

The CT images in arterial and venous phases were isotropically resampled by using trilinear interpolation in Artificial Intelligence Kit software (A. K, version: 3.3.0. R, GE Healthcare, USA) with a voxel size of 1 × 1 × 1 mm to minimize the effect of different scanning protocols or equipment on the radiomics features [29]. Region of interest (ROI) segmentation was performed by delineating around the tumor outline for the largest cross-sectional area in the CT axial plane (Fig. 1). Care was taken to avoid the gastric cavity and stomach contents, fatty tissue around the stomach wall and blood vessels when segmenting. Each ROI was outlined by a radiologist (L.C. 6 years of experience in abdominal imaging diagnosis) and supervised by a radiologist (Z.H., 8 years of experience in abdominal imaging diagnosis). To ensure the reliability and reproducibility of the radiomics features, 30 patients were randomly selected for their data to be segmented. For an analysis of interobserver agreement, a radiologist (L. CC) conducted the first-time whole-dataset segmentation, and another radiologist (H.W., 7 years of experience in abdominal imaging diagnosis) who was supervised by a radiologist (L.L., 9 years of experience in abdominal imaging diagnosis) delineated the images of the 30 selected patients during the same period. For analysis of intraobserver agreement, the radiologist (L. CC) repeatedly conducted segmentation 1 month after the first delineation.

### Radiomics feature extraction

The radiomics features were automatically extracted by using the Python package Pyradiomics [30]. A total of 1409 radiomics features were separately extracted from the delineated ROI in the arterial and venous phases. There were 107 features extracted from the original images, including 32 first-order features (18 intensity statistical and 14 shape features). Among the 75 textural features, there were 24 gray-level cooccurrence matrix (GLCM), 16 gray-level run length matrix (GLRLM), 16 gray-level size zone matrix (GLSZM), 14 gray-level dependence matrix (GLDM) and 5 neighboring gray tone difference matrix (NGTDM) features. In addition, the same number of first-order grayscale statistical features and texture features were extracted based on different transformed images. A total of 744 features were extracted based on wavelet decomposition images with 8 filter channels, 279 features were extracted based on Laplacian of Gaussian (LoG) transform images (sigma parameters selected as 1.0 mm, 3.0 mm and 5.0 mm), and 279 features were extracted based on local binary pattern (LBP)-filtered images (2nd-order spherical harmonic function, spherical neighborhood operator with radius 1.0 and fine fraction 1) [30]. The features were extracted by discretizing the CT values of the ROI region based on a fixed interval width (bin width = 25 HU). Then, the intra/interclass correlation coefficients (ICCs) were calculated based on the features extracted from the 30 randomly selected patients. The features with intra- and interobserver ICC values simultaneously greater than 0.7 were retained for assessment of arterial- and venous-phase features, respectively [31].

### Radiomics feature selection and model establishment

The training dataset was used for feature selection and modeling, and the same procedure and set of parameters were applied to the external dataset for model validation.

The same method described above was used to perform feature preprocessing and feature screening in the arterial and venous phases and to build independent arterial and venous radiomics models. The feature selection and modeling were performed as follows.

1. Outlier processing occurred with values greater than the third quartile + 2 × quartile distance being converted to the 95th percentile; values less than the first quartile − 2 × quartile distance were converted to the 10th percentile.
2. Features with relatively low variance values less than 1.0 were excluded.
3. Missing data were filled with the median value, and the Z Score standardization method was applied for data standardization and normalization.
4. The less redundant features were retained by using correlation analysis with a cutoff value of 0.9.
5. Features with importance coefficients greater than the maximum importance coefficient/3 assessed using the gradient-boosted decision trees (GBDT) feature importance ranking based on decision tree methods were retained.
6. Radiomics models were established using the naïve Bayes classifier, and the predicted probability of the model output was used as the Radscore for each individual model.

Based on the above feature screening and modeling methods, our study developed two radiomics models based on pCR outcome: (1) an arterial radiomics model with Radscore^_AP_pCR^ and (2) a venous radiomics model with Radscore^_VP_pCR^.

### Clinical feature selection and model establishment

The clinical features were screened using GBDT (selection of the top three features of importance) and univariate logistic regression (*p* < 0.1). Model building was performed using the naïve Bayes classifier, and the predicted probability of the model output was used as the model score of the clinical model. Based on the above feature screening and modeling methods, clinical models for predicting pCR (Score^_clinic_pCR^) were established.

### Combined model establishment

Based on the established Radscores and clinical factors, radiomics–clinical combined models were developed using the naïve Bayes classifier. Four combined models were built, including the following: (1) arterial–venous combined model; (2) arterial–clinical combined model; (3) venous–clinical combined model; and (4) arterial–venous–clinical combined model.

### Evaluation of model predictive performance

The performance of the model was evaluated by using receiver operating characteristic curve (ROC) analysis to obtain the area under the ROC curve (AUC). The sensitivity, accuracy, negative predictive value and positive predictive value were calculated from the cutoff values of the model score corresponding to the maximum Youden index to evaluate the discriminatory performance of the model. The cutoff value of the training group data was applied to the validation group data to obtain their corresponding discriminatory efficacies in the external validation group. The calibration ability of the model was mainly tested using calibration curve analysis and the Hosmer–Lemeshow test for goodness of fit (*p* > 0.05 indicates no significant difference between the predicted and actual values). To compare the AUC of different models, Delong’s test was applied (*p* < 0.05 indicates a significant difference). A decision curve analysis (DCA) was used to assess the net clinical benefit or clinical utility obtained by the model at different threshold probabilities.

### Statistical analysis

Statistical analysis was performed using R software (version 3.6.3, http://www.r-project.org). Continuous variable comparisons between two groups were made using independent samples *t* tests (for data conforming to a normal distribution) or Mann–Whitney *U* tests (for data not conforming to a normal distribution). Categorical variables were tested by the chi-square test or Fisher's exact test. A two-sided *p* value of < 0.05 was considered statistically significant. The following R packages were applied: “icc” for intra/interclass correlation coefficient; “glmnet” for logistic regression; “pROC” for ROC analysis; “rmda” for DCA; calibration function in the “rms” package for calibration analysis, “gbm” for GBDT feature importance analysis, “naïveBayes” function for Naïve Bayes classifier, “adabag” package for AdaBoost classifier, “e1071” package for SVM classifier, and “rpart” package for decision tree classifier.

## Results

### Model establishment for predicting pCR

#### Arterial model for predicting pCR

**Fig. 2:**
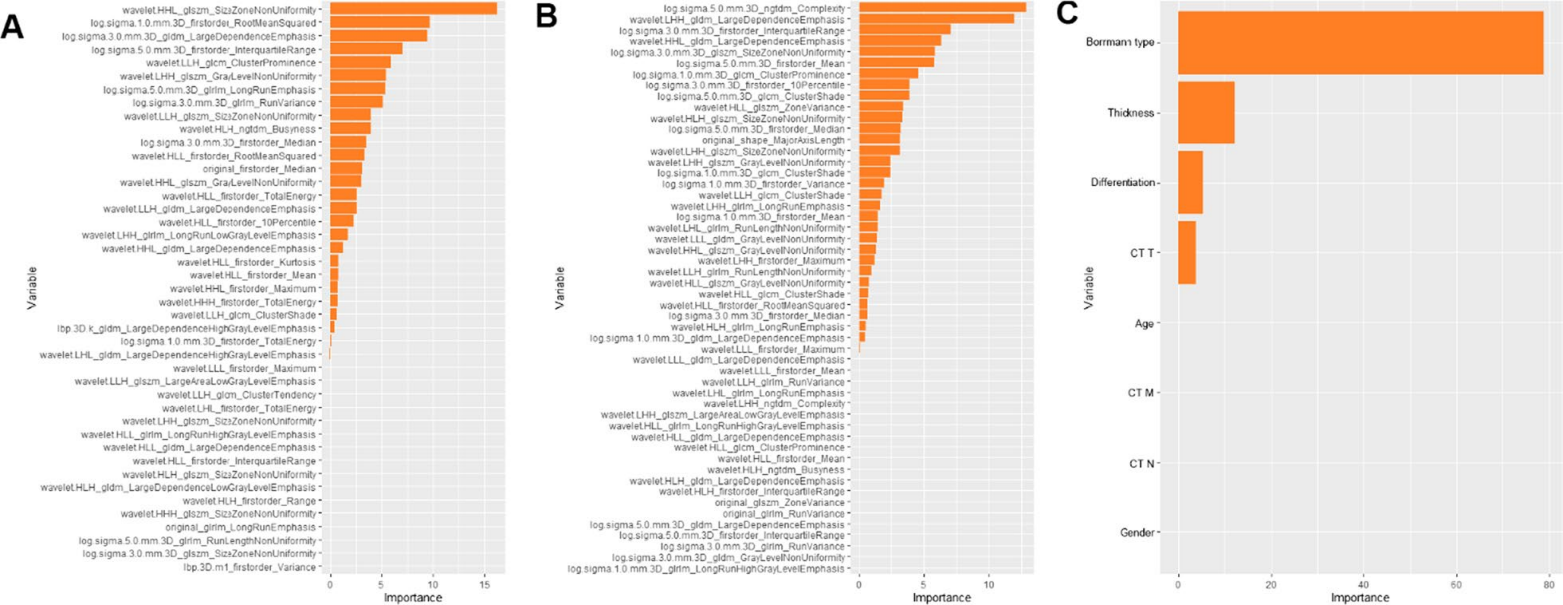
The ranked importance of GBDT selected features for each independent model. **A** Arterial-phase radiomics features. **B** The venous-phase radiomics features. **C** The clinical factors

By using intra/interclass consistency analysis, 395 radiomics features with ICCs > 0.70 were retained among 1409 features. After removing features with a variance less than 1.0, 150 features were retained. Then, 43 features were retained after correlation analysis by using a cutoff value of 0.9. Six features were retained by GBDT feature importance ranking (Fig. 2A), and the arterial model was established using the naïve Bayes classifier. The AUC values and 95% confidence intervals for the analysis of AEG using single arterial features for predicting pCR in the training and external validation groups are summarized in Additional file1: Table S1, and the ROC curves are shown in Additional file1: Fig. S1A, B. The arterial model radiomics features and the differences in Rad-score^AP_pCR^ in the training and external validation groups are summarized in Additional file1: Table S2.

#### Venous model for predicting pCR

By using intra/interclass consistency analysis, 410 radiomics features with ICCs > 0.70 were retained among 1409 features. After removing features with variance less than 1.0, 165 features were retained. Then, 52 features were retained after correlation analysis by using a cutoff value of 0.9. Seven features were retained by GBDT feature importance ranking (Fig. 2B), and the venous model was established using the naïve Bayes classifier. The AUC values and 95% confidence intervals for the analysis of AEG using single venous features for predicting pCR in the training and external validation groups are shown in Additional file1: Table S3, and the ROC curves are shown in Additional file1: Fig. S1C, D. The venous model radiomics features and the differences in Rad-score^VP_pCR^ in the training and validation groups are shown in Additional file1: Table S4.

#### Clinical model for predicting pCR

The results of the GBDT feature importance ranking are plotted in Fig. 2C, and the univariate logistic regression results for the clinical factors are summarized in Additional file1: Table S5. The Borrmann classification, tumor thickness, and differentiation degree were finally selected and incorporated into the naïve Bayes classifier to establish the clinical model.

### The evaluation of model performance

**Fig. 3:**
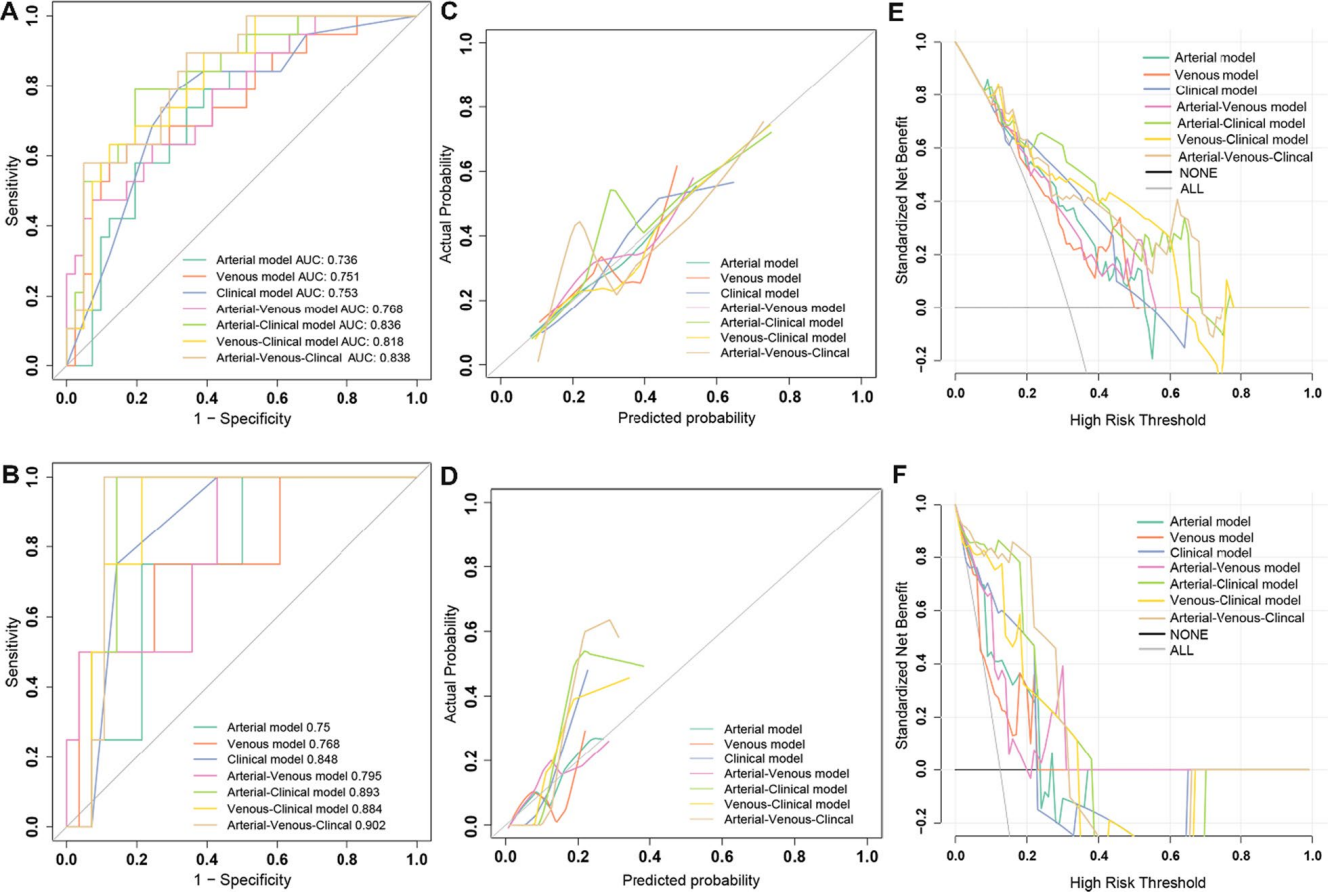
The performance of different models. **A**, **B** ROC curves of different models for predicting pathological complete response (pCR) of adenocarcinoma of the esophagogastric junction (AEG) in the training group (**A**) and external validation group (**B**). **C**, **D** Calibration curves of different models predicting pCR in the training group (**C**) and external validation group (**D**). The 45-degree sloping line indicates the ideal calibration, and the closer the model calibration curve is to the ideal calibration line, the better the agreement between the model predicted probability and the actual probability. **E**, **F** Decision curves of different prediction models in the training group (**E**) and external validation group (**F**). The X-axis is the threshold probability range, and the Y-axis is the net benefit. The black line labeled "NONE" indicates that no lesions are assumed to be pCR, and the gray line labeled "ALL" indicates that all lesions are assumed to be pCR. The further away from both the black and gray lines, the higher the net benefit of the model compared to performance utilizing the "NONE" and "ALL" assumptions. When comparing the decision curves of different models within the same range of threshold probability, the larger the area under the curve for the same threshold probability interval, the higher the net benefit of the model at that threshold probability

The AUC values, specificity, sensitivity, accuracy, positive predictive value and negative predictive value of the seven models developed in this study for predicting pCR in the training and external validation groups are shown in Table 2. The ROC curves are shown in Fig. 3A, B. The results of the Delong test used to compare the AUC values of the seven models in the training and validation groups are shown in Additional file1: Table S6. The calibration curves were used to visualize the calibration of all models (Fig. 3C, D), the Hosmer–Lemeshow test was used to assess the goodness of fit (Additional file1: Table S7), and the decision curves were used to confirm the clinical usefulness of the models (Fig. 3E, F).

**Table 2:** Efficacy of different models in the training group and the external validation group for predicting pathological complete response (pCR)

Model	Arterial model	Venous model	Clinical model	Arterial–venous combined model	Arterial–clinical combined model	Venous–clinical combined model	Arterial–venous–clinical combined model
*Training group*							
AUC	0.736	0.751	0.753	0.768	0.836	0.818	0.838
95%CI	0.607–0.865	0.614–0.888	0.622–0.884	0.639–0.896	0.728–0.943	0.708–0.927	0.736–0.941
Threshold	0.510	0.856	0.351	0.667	0.332	0.543	0.160
Specificity	0.610	0.829	0.683	0.927	0.805	0.878	0.659
Sensitivity	0.789	0.632	0.789	0.474	0.789	0.632	0.895
Accuracy	0.667	0.767	0.717	0.783	0.800	0.800	0.733
NPV	0.862	0.829	0.875	0.792	0.892	0.837	0.931
PPV	0.484	0.632	0.536	0.750	0.652	0.706	0.548
*External validation group*							
AUC	0.750	0.768	0.848	0.795	0.893	0.884	0.902
95%CI	0.535–0.965	0.489–1	0.710–0.987	0.560–1	0.780–1	0.762–1	0.792–1
Threshold	0.510	0.856	0.351	0.667	0.332	0.543	0.160
Specificity	0.536	0.750	0.857	0.857	0.786	0.893	0.500
Sensitivity	0.750	0.750	0.750	0.500	1	0.500	1
Accuracy	0.562	0.750	0.844	0.812	0.812	0.844	0.562
NPV	0.938	0.955	0.960	0.923	1	0.926	1
PPV	0.188	0.300	0.429	0.333	0.400	0.400	0.222

To explore the reliability of the developed model, we also used other modeling methods (logistic regression, support vector machine, decision tree, AdaBoost) for the establishment of a pCR radiomics model and a clinical model based on the same feature sets, and the results are shown in Additional file1: Tables S8–S10. These results indicated that the Naïve Bayes classifier could achieve better model performance in the radiomics and clinical models.

## Discussion

In this retrospective study, we analyzed the clinical, pathological and enhanced CT imaging data of patients with advanced AEG before NAC at two medical centers to develop radiomics, clinical, and combined models for predicting pathologically determined TRG grading. The results showed that the combined radiomics–clinical model has the potential to be a reference for evaluating tumor response and developing treatment strategies for patients with AEG.

Preoperative NAC has been shown to reduce the stage of AEG, increase the rate of radical surgical resection, and improve the long-term prognosis of patients [2–5, 11, 32]. However, 40–70% of patients have a poor response to NAC. Some patients miss the best opportunity for direct operation due to tumor progression during the process of NAC [33, 34]. Therefore, it is clinically relevant to monitor the efficacy of NAC during treatment and to identify patients who respond to NAC in a timely and accurate manner. Radiomics integrates meaningful quantitative imaging features for modeling, which is a major difference from methods utilizing the traditional visual interpretation of images [35–39]. Chen et al. [40] used the features of the CT venous phase and established a predictive model to distinguish between advanced gastric cancer patients with potentially pathologically significant reactions and those with mild reactions and were able to effectively stratify patients according to their response to NAC. Mazzei et al. [41] performed a multicenter study to predict NAC response by delta radiomics in locally progressive gastric cancer. The only significant TA variable was the delta gray-level cooccurrence matrix (GLCM) contrast, with a model AUC value of 0.763. However, there are few reports on the predicted efficacy of NAC for AEG alone. Therefore, we attempted to establish prediction models that help decision-making for the development of individualized and precise treatment strategies for AEG patients.

In this study, 6 and 7 potential radiomics features were selected following screening of the arterial and venous phase images, respectively, and the types of features that appeared in both the arterial and venous phases were LargeDependenceEmphasis (GLDM), InterquartileRange (first-order) and SizeZoneNonUniformity (GLSZM). The GLDM matrix describes the dependence of the voxel intensity. The LargeDependenceEmphasis feature had higher values in the pCR group, while the InterquartileRange and SizeZoneNonUniformity features had lower values in the pCR group. These feature characteristics might indicate that smaller grayscale distribution intervals and a more uniform grayscale distribution of the lesion may suggest the possibility of pCR. It has also been reported that lower GLSZM variants, which represent lower tumor heterogeneity, may be useful in assessing the response to bevacizumab treatment in colorectal cancer [42].

More features were retained in the venous phase images than in the arterial phase images. When the tumor regressed after NAC, the new capillaries and proliferating fibroblasts with rich blood supply were transformed into scar tissue and fibrous connective tissue, and the blood supply in the tissues was reduced. Therefore, venous phase images can reflect the blood supply characteristics of AEG more clearly and comprehensively. In future studies, venous phase images can be selected as the source of radiomics features.

In the pCR-based prediction model, the AUC values obtained using the venous model were higher than those obtained using the arterial model in both the training and external validation groups (0.751 vs. 0.736, 0.768 vs. 0.750), which was consistent with the fact that the venous phase images reflect the AEG blood supply more clearly and comprehensively. Among all clinical characteristics analyzed, the Borrmann type, tumor thickness, and degree of tumor differentiation had predictive value for NAC efficacy. The combination of multiphase radiomics and clinical factors could be used to further improve the discriminatory performance beyond that achieved with independent models. When the Rad-score^AP_pCR^, Rad-score^VP_pCR^ and clinical factors were combined simultaneously, an AUC value of 0.838 was achieved in the training cohort and a value of 0.902 was achieved in the external validation cohort. The potential reasons for the good performance of the combined model may be related to the intratumoral heterogeneity reflected by the radiomics features and clinical features that describe the biological behavior of the tumor as well as the cell cycle regulation and chemokine signaling that are occurring, which are important factors affecting the efficacy of NAC [43]. Several studies have reported that tumors with greater heterogeneity tend to be more aggressive in terms of proliferation, metastasis and angiogenesis and may be more resistant to NAC [44, 45]. The complete mechanism underlying the correlation between radiomics features and NAC response has not been elucidated, and studies utilizing radiogenomics are necessary to provide evidence in addressing this issue [46], including studies on the characteristics and the textural features of responsive lesions and the composition of the solid region of the tumor and the surrounding infiltrative environment, which can be described by quantitative radiomics features.

The current study demonstrated the potential use of enhanced CT radiomics features for predicting pCR after NAC in AEG patients. The external validation of the established model also guarantees the reliability of the model performance. It is worth noting that there are some remaining limitations of this study. First, the CT images were obtained from different CT scanners, and there were some differences in the CT scanning protocols of the patients. However, we minimized these differences to the greatest extent by resampling the CT images of the enrolled patients and normalizing the extracted radiomics features. Standardized scanning protocols need to be uniformly applied in future prospective studies. Second, for TRG classification, some patients with progressive disease after NAC could not be operated on and were not included in our study. Because the NAC protocol has not yet been unified, in our study, patients who underwent the XELOX protocol and SOX protocol were selected, which is consistent with the actual clinical treatment situation at present but also introduces a certain selection bias. In addition, our study only included data from two centers, the sample size was still small, the molecular typing of AEG was not performed, and the effect of genes on NAC was not considered. The naïve Bayes classifier selected in the current study is a probabilistic classifier that is suitable for a small dataset. Large sample studies combined with genetic information are needed to further improve and validate the testing efficacy and generalizability of the model. A predictive model with better efficacy that could be translated into clinical practice is needed.

## Conclusion

In conclusion, a radiomics method based on enhanced CT imaging before NAC was validated as a potential method for predicting whether advanced AEG patients could achieve pCR after NAC in our study. The combination of radiomics and clinical factors can be used to effectively improve the predictive performance. The prediction model established in our study can be used to stratify patients according to their potential responsiveness to NAC, which can help provide a basis for clinicians to develop individualized and precise treatment plans.

## Supplementary Information

**Additional file 1**. Radiomics features predict performance of neoadjuvant chemotherapy.

## Data Availability

The dataset used or analyzed during the current study is available from the corresponding author on reasonable request.

## Abbreviations

AEG: Adenocarcinoma of the esophagogastric junction
AJCC: American Joint Committee on Cancer
AUC: Under the ROC curve
CA125: Carbohydrate antigen 125
CA199: Carbohydrate antigen 199
CEA: Carcinoembryonic antigen
DCA: Decision curve analysis
EGJ: Esophagogastric junction
GBDT: The gradient-boosted decision trees
GLCM: Gray-level co-occurrence matrix
GLDM: Gray-level dependence matrix
GLRLM: Gray-level run length matrix
GLSZM: Gray-level size zone matrix
NAC: Neoadjuvant chemotherapy
NGTDM: Neighboring gray tone difference matrix
pCR: Pathological complete response
RECIST: Response evaluation criteria in solid tumors
ROC: Receiver operating characteristic curve
TRG: Tumor regression grade
