# Supplementary material for: Enhanced CT-based radiomics predicts pathological complete response after neoadjuvant chemotherapy for advanced adenocarcinoma of the esophagogastric junction: a two-center study
Source: Insights Imaging. 2022 Aug 17;13:134. doi: 10.1186/s13244-022-01273-w (PMC9385906; doi:10.1186/s13244-022-01273-w)
Supplement: Supplementary file 1 — Additional file 1. Radiomics features predict performance of neoadjuvant chemotherapy. [file 13244_2022_1273_MOESM1_ESM.pdf]

ELECTRONIC SUPPLEMENTARY MATERIAL

Enhanced CT-based radiomics predicts pathological complete response after neoadjuvant chemotherapy for advanced adenocarcinoma of the esophagogastric junction: A two-center study

Table S1. AUC values and 95% confidence intervals for selected arterial radiomics features in predicting the pCR of AEG in the training and external validation groups

	Training group		External validation group	
Variable	AUC values	95%CI	AUC values	95%CI
wavelet.HHL_glszm_SizeZoneNonUniformity	0.606	0.453～0.759	0.728	0.352～1.000
log.sigma.1.0.mm.3D_firstorder_RootMeanSquared	0.583	0.431～0.736	0.634	0.382～0.886
log.sigma.3.0.mm.3D_gldm_LargeDependenceEmphasis	0.629	0.486～0.772	0.580	0.374～0.787
log.sigma.5.0.mm.3D_firstorder_InterquartileRange	0.615	0.472～0.758	0.616	0.307～0.925
wavelet.LLH_glcm_ClusterProminence	0.579	0.415～0.743	0.598	0.384～0.813
wavelet.LHH_glszm_GrayLevelNonUniformity	0.603	0.448～0.758	0.482	0.166～0.799

Note: CI: confidential interval; pCR: pathological complete response; AEG: adenocarcinoma of the esophagogastric junction

Table S2. Statistical differences of the selected arterial radiomics features and Rad-score–AP–pCR between pCR and non-pCR groups in the training and external validation groups

Training group					
Variable	Sample	pCR	Non-pCR	Statistics	*P* value
wavelet.HHL_glszm_SizeZoneNonUniformity	60	-0.43(-0.51, -0.11)	-0.48(-0.52, -0.37)	1.311	0.190
log.sigma.1.0.mm.3D_firstorder_RootMeanSquared	60	-0.19(-0.64, 0.43)	-0.27(-0.71, -0.18)	1.033	0.302
log.sigma.3.0.mm.3D_gldm_LargeDependenceEmphasis	60	-0.14±1.09	0.31±0.69	-1.915	0.061
log.sigma.5.0.mm.3D_firstorder_InterquartileRange	60	-0.18(-0.67, 1.12)	-0.47(-0.68, -0.09)	1.422	0.155
wavelet.LLH_glcm_ClusterProminence	60	-0.35(-0.37, -0.25)	-0.37(-0.37, -0.30)	0.977	0.328
wavelet.LHH_glszm_GrayLevelNonUniformity	60	-0.25(-0.75, 0.05)	0.01(-0.54, 0.50)	-1.279	0.201
Rad-score–AP–pCR	60	0.35(0.00, 0.66)	0.68(0.53, 0.76)	-2.916	0.004
External validation group					
Variable	Sample	pCR	Non-pCR	Statistics	*P* value
wavelet.HHL_glszm_SizeZoneNonUniformity	32	-0.37(-0.48, -0.16)	-0.51(-0.57, -0.17)	1.453	0.146
log.sigma.1.0.mm.3D_firstorder_RootMeanSquared	32	-0.74(-1.20, 0.13)	-1.18(-1.23, -0.68)	0.855	0.393

log.sigma.3.0.mm.3D_gldm_LargeDependenceEmphasis	32	0.62(-0.75, 1.28)	0.82(0.43, 0.98)	-0.513	0.608
log.sigma.5.0.mm.3D_firstorder_InterquartileRange	32	-0.56(-0.90, 0.69)	-0.65(-1.05, -0.41)	0.741	0.459
wavelet.LLH_glcm_ClusterProminence	32	-0.37(-0.37, -0.34)	-0.37(-0.37, -0.35)	-0.627	0.531
wavelet.LHH_glszm_GrayLevelNonUniformity	32	-0.26(-0.65, 0.22)	-0.10(-0.67, 0.11)	0.114	0.909
Rad-score^{AP_pCR}	32	0.48(0.06, 0.70)	0.74(0.59, 0.77)	-1.595	0.111

Note: pCR: pathological complete response; The variables without normal distribution were depicted by median (interquartile range, IQR); The variables with normal distribution were depicted by mean ± SD; Statistically significant level: $P < 0.05$

Table S3. AUC values and 95% confidence intervals for selected venous radiomics features in predicting the pCR of AEG in the training and external validation groups

	Training group		External validation group	
Variable	AUC values	95%CI	Variable	AUC values
log.sigma.5.0.mm.3D_ngtdm_Complexity	0.674	0.521～0.827	0.643	0.400～0.886
wavelet.LHH_gldm_LargeDependenceEmphasis	0.634	0.480～0.788	0.545	0.267～0.823
log.sigma.3.0.mm.3D_firstorder_InterquartileRange	0.648	0.499～0.797	0.563	0.335～0.790
wavelet.HHL_gldm_LargeDependenceEmphasis	0.588	0.419～0.757	0.679	0.370～0.988

log.sigma.3.0.mm.3D_glszm_SizeZoneNonUniformity	0.638	0.492～0.784	0.638	0.437～0.840
log.sigma.5.0.mm.3D_firstorder_Mean	0.562	0.418～0.706	0.696	0.471～0.922
log.sigma.1.0.mm.3D_glcm_ClusterProminence	0.640	0.484～0.796	0.741	0.436～1.000

Note: CI: confidential interval; pCR: pathological complete response; AEG: adenocarcinoma of the esophagogastric junction

Table S4. Statistical differences of the selected venous radiomics features and Rad-score$_{-}^{VP_pCR}$ between pCR and non-pCR groups in the training and external validation groups

Training group					
Variable	Sample	pCR	Non-pCR	Statistics	*P* value
log.sigma.5.0.mm.3D_ngtdm_Complexity	60	-0.32(-0.70, 0.76)	-0.67(-0.83, -0.36)	2.153	0.031
wavelet.LHH_gldm_LargeDependenceEmphasis	60	0.38(-0.48, 0.75)	-0.12(-1.04, 0.53)	1.661	0.097
log.sigma.3.0.mm.3D_firstorder_InterquartileRange	60	-0.01(-0.48, 0.79)	-0.47(-1.02, 0.24)	1.835	0.066
wavelet.HHL_gldm_LargeDependenceEmphasis	60	0.19(-0.21, 0.52)	0.55(-0.09, 0.64)	-1.089	0.276
log.sigma.3.0.mm.3D_glszm_SizeZoneNonUniformity	60	-0.45(-0.68, 0.76)	-0.53(-0.75, -0.35)	1.708	0.088
log.sigma.5.0.mm.3D_firstorder_Mean	60	0.21(-0.64, 0.81)	0.47(-0.14, 0.65)	-0.771	0.441

log.sigma.1.0.mm.3D_glcm_ClusterProminence	60	-0.46(-0.54, 0.07)	-0.54(-0.55, -0.32)	1.732	0.083
Rad-score^{_VP_pCR}	60	0.60(0.00, 0.82)	0.87(0.62, 0.93)	-3.107	0.002

External validation group

Variable	Sample	pCR	Non-pCR	Statistics	*P* value
log.sigma.5.0.mm.3D_ngtdm_Complexity	32	-0.59(-0.82, 0.40)	-0.80(-0.84, -0.45)	0.912	0.362
wavelet.LHH_gldm_LargeDependenceEmphasis	32	-0.44±0.73	-0.63±0.59	0.489	0.628
log.sigma.3.0.mm.3D_firstorder_InterquartileRange	32	-0.66(-1.15, 0.04)	-0.96(-1.04, -0.54)	0.399	0.690
wavelet.HHL_gldm_LargeDependenceEmphasis	32	-0.19(-0.53, 0.04)	-0.41(-0.92, -0.13)	1.140	0.254
log.sigma.3.0.mm.3D_glszm_SizeZoneNonUniformity	32	-0.64(-0.75, -0.12)	-0.41(-0.62, -0.19)	-0.883	0.377
log.sigma.5.0.mm.3D_firstorder_Mean	32	-0.02±1.11	0.71±0.56	-2.076	0.077
log.sigma.1.0.mm.3D_glcm_ClusterProminence	32	-0.55(-0.56, -0.41)	-0.57(-0.57, -0.53)	1.538	0.124
Rad-score_VP_pCR	32	0.70(0.16, 0.88)	0.92(0.65, 0.94)	-1.709	0.087

Note: pCR: pathological complete response; The variables without normal distribution were depicted by median (interquartile range, IQR); The variables with normal distribution were depicted by mean ± SD; Statistically significant level: $P < 0.05$

Table S5. The results of univariate logistic regression analysis of clinical characteristics in predicting pCR of AEG patients

Variable	B	Wald	Odds ratio	95% CI	*P* value

Gender	-18.109	0	0	NA～9.303×10^{54}	0.993
Age	-0.583	0.910	0.558	0.156～1.778	0.340
T-staging before NAC	-0.884	2.391	0.413	0.129～1.242	0.122
N-staging before NAC	-0.327	0.309	0.721	0.216～2.232	0.578
Borrmann typing	-1.667	6.658	0.189	0.047～0.624	0.010
CEA	-1.124	2.522	0.325	0.068～1.179	0.112
CA199	0.903	1.373	2.467	0.523～11.718	0.241
CA125	-1.088	1.076	0.337	0.018～1.757	0.300
Serum albumin	0.564	1.008	1.757	0.589～5.427	0.315
Tumor thickness	1.275	4.386	3.578	1.137～12.812	0.036
Degree of tumor differentiation	0.975	2.932	2.652	0.879～8.361	0.087

Note: B logistic regression coefficient; CI confidence interval; Statistically significant level: $P < 0.05$

Table S6. The results of Delong test for AUC values of all models

	Training group		External validation group	
Models	*Z*	*P* value	*Z*	*P* value
Arterial model.vs. Venous model	-0.180	0.857	-0.088	0.930
Arterial model.vs. Clinical model	-0.175	0.861	-0.668	0.504
Arterial model.vs. Arterial-Venous combined model	-0.694	0.488	-0.418	0.676
Arterial model.vs. Arterial-Clinical combined model	-1.481	0.139	-1.294	0.196
Arterial model.vs. Venous-Clinical combined model	-0.959	0.338	-1.037	0.300
Arterial model.vs. Arterial-Venous-Clinical combined model	-1.657	0.097	-1.300	0.193
Venous model.vs. Clinical model	-0.019	0.985	-0.517	0.605
Venous model.vs. Arterial-Venous combined model	-0.303	0.762	-0.202	0.840
Venous model.vs. Arterial-Clinical combined model	-0.964	0.335	-0.797	0.426
venous model.vs.Venous-Clinical combined model	-0.955	0.340	-0.981	0.327
venous model.vs. Arterial-Venous-Clinical combined model	-1.305	0.192	-0.925	0.355
Clinical model.vs. Arterial-Venous combined model	-0.150	0.881	0.347	0.729
Clinical model.vs. Arterial-Clinical combined model	-1.957	0.050	-0.963	0.336

Clinical model.vs. Venous-Clinical combined model	-1.593	0.111	-0.561	0.575
Clinical model.vs. Arterial-Venous-Clinical combined model	-1.436	0.151	-1.093	0.275
Arterial-Venous combined model.vs. Arterial-Clinical combined model	-0.965	0.335	-0.771	0.441
Arterial-Venous combined model.vs. Venous-Clinical combined model	-0.680	0.496	-0.819	0.413
Arterial-Venous combined model.vs. Arterial-Venous-Clinical combined model	-1.348	0.178	-0.897	0.370
Arterial-Clinical combined model.vs. Venous-Clinical combined model	0.450	0.652	0.169	0.866
Arterial-Clinical combined model.vs. Arterial-Venous-Clinical combined model	-0.084	0.933	-0.337	0.736
Venous-Clinical combined model.vs. Arterial-Venous-Clinical combined model	-0.593	0.553	-0.500	0.617

Table S7. The results of the Hosmer-Lemeshow test for different models in the training and external validation groups

	Training group		External validation group	
Model	Statistics	*P* value	Models	Statistics
Arterial model	4.997	0.758	7.949	0.438

Venous model	8.427	0.393	8.467	0.389
Clinical model	0.732	0.392	1.189	0.276
Arterial-Venous combined model	12.152	0.145	5.326	0.722
Arterial-Clinical combined model	6.900	0.547	1.831	0.986
Venous-Clinical combined model	11.885	0.156	2.038	0.980
Arterial-Venous-Clinical combined model	14.879	0.062	10.478	0.233

Note: *P*>0.05: no significant difference between the predicted and actual value.

Table S8. Performance of arterial models based on other classifiers in the training and external validation groups

	Model	AUC	95%CI	Threshold	Specificity	Sensitivity	Accuracy	NPV	PPV
Training group	Naïve Bayes	0.736	0.607～0.865	0.510	0.610	0.789	0.667	0.862	0.484
	Logistic regression	0.720	0.592～0.848	0.341	0.634	0.789	0.683	0.867	0.500
	Support vector machines	0.707	0.564～0.850	1.000	0.732	0.632	0.700	0.811	0.522
	Decision tree	0.856	0.768～0.945	0.588	0.756	0.895	0.800	0.939	0.630
	AdaBoost	1.000	1.000～1.000	0.493	1.000	1.000	1.000	1.000	1.000

External validation group	Naïve Bayes	0.750	0.535～0.965	0.510	0.536	0.750	0.563	0.938	0.188
	Logistic regression	0.679	0.417～0.940	0.341	0.536	0.750	0.563	0.938	0.188
	Support vector machines	0.554	0.315～0.792	1.000	0.643	0.250	0.594	0.857	0.091
	Decision tree	0.652	0.426～0.870	0.588	0.500	0.750	0.531	0.933	0.176
	AdaBoost	0.607	0.372～0.842	0.493	0.821	0.250	0.750	0.885	0.167

Table S9. Performance of venous models based on other classifiers in the training and external validation groups

	Model	AUC	95%CI	Threshold	Specificity	Sensitivity	Accuracy	NPV	PPV
Training group	Naïve Bayes	0.751	0.614～0.888	0.856	0.829	0.632	0.767	0.829	0.632
	Logistic regression	0.728	0.581～0.875	0.396	0.854	0.579	0.767	0.814	0.647
	Support vector machines	1.000	1.000～1.000	0.042	1.000	1.000	1.000	1.000	1.000
	Decision tree	0.367	0.254～0.479	/	0	1.000	0.317	/	0.317
	AdaBoost	1.000	1.000～1.000	0.493	1.000	1.000	1.000	1.000	1.000

	Model	AUC	95%CI	Threshold	Specificity	Sensitivity	Accuracy	NPV	PPV
External validation group	Naïve Bayes	0.768	0.489～1.000	0.856	0.750	0.750	0.750	0.955	0.300
	Logistic regression	0.491	0.212～0.770	0.396	0.429	0.500	0.438	0.857	0.111
	Support vector machines	0.527	0.179～0.874	0.042	0	1.000	0.125	/	0.125
	Decision tree	0.589	0.293～0.886	/	1.000	0	0.875	0.875	/
	AdaBoost	0.759	0.414～1.000	0.493	0.821	0.750	0.813	0.958	0.375

Table S10. Performance of clinical models based on other classifiers in the training and external validation groups

	Model	AUC	95%CI	Threshold	Specificity	Sensitivity	Accuracy	NPV	PPV
Training group	Naïve Bayes	0.753	0.622～0.884	0.351	0.683	0.789	0.717	0.875	0.536
	Logistic regression	0.753	0.622～0.884	0.364	0.683	0.789	0.717	0.875	0.536
	Support vector machines	0.750	0.619～0.881	0.999	0.756	0.684	0.733	0.838	0.565
	Decision tree	0.733	0.603～0.863	0.606	0.756	0.684	0.733	0.838	0.565
	AdaBoost	0.791	0.680～0.903	0.340	0.610	0.895	0.700	0.926	0.515

External validation group	Naïve Bayes	0.848	0.710～0.987	0.351	0.857	0.750	0.844	0.960	0.429
	Logistic regression	0.848	0.710～0.987	0.364	0.857	0.750	0.844	0.960	0.429
	Support vector machines	0.304	0.187～0.420	0.999	0.071	1.000	0.188	1.000	0.133
	Decision tree	0.723	0.473～0.973	0.606	0.929	0	0.813	0.867	0
	AdaBoost	0.670	0.490～0.849	0.340	0.679	0.750	0.688	0.950	0.250

Figure:

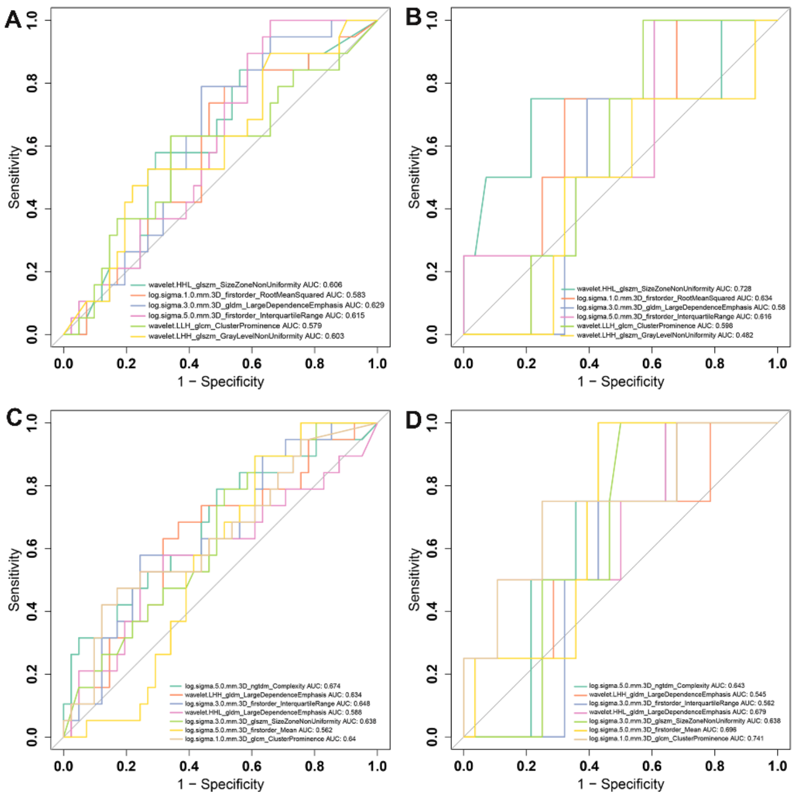

Fig.S1 ROC curves of individual features in different models to predict pathological complete response (pCR). 1A: ROC curves of individual features of arterial model predicting pCR in the training group; 1B: ROC curves of individual features of arterial model predicting pCR in the external validation group; 1C: ROC curves of individual features of venous model predicting pCR in the training group; 1D: ROC curves of individual features of venous model predicting pCR in the external validation group.
